# Fever, Frustration and Fortitude: A Qualitative Exploration of Nursing Practice in Paediatric Settings

**DOI:** 10.1002/nop2.70728

**Published:** 2026-07-31

**Authors:** Zahra Sarkoohi, Jamileh Farokhzadian, Maryam Askaryzadeh Mahani, Monirsadat Nematollahi

**Affiliations:** ^1^ Student Research Committee Kerman University of Medical Sciences Kerman Iran; ^2^ Nursing Research Center Kerman University of Medical Sciences Kerman Iran; ^3^ Department of Nursing, School of Nursing and Midwifery Bam University of Medical Sciences Bam Iran; ^4^ Reproductive and family health research center Kerman University of Medical Sciences Kerman Iran

**Keywords:** nurses' experiences, nurses' perceptions, paediatric fever, qualitative content analysis

## Abstract

**Background:**

Fever is one of the most common symptoms in children and a leading cause of their hospitalisation. Nurses, as primary caregivers in paediatric wards, play a pivotal role in the assessment and management of children with fever. However, nurses' perceptions and experiences of fever management are influenced by cultural contexts, psychological and social factors, as well as prevailing health system policies.

**Aim:**

The aim of the study was to explore nurses' perceptions influencing their management of childhood fever, including barriers and facilitators to evidence‐based practice.

**Design:**

A qualitative descriptive design using a conventional content analysis approach.

**Methods:**

Nineteen nurses working in paediatric wards participated in individual semi‐structured interviews conducted between March 20, 2024 and May 20, 2025. Purposive sampling was used to recruit participants with direct experience in managing fever in children. Data were audio‐recorded, transcribed verbatim and analysed inductively using qualitative content analysis as described by Graneheim and Lundman. The study adhered to the Consolidated Criteria for Reporting Qualitative Research (COREQ) guidelines.

**Results:**

Data analysis resulted in the extraction of one main theme titled ‘Navigating Uncertainty and Psychological Strain in Pediatric Fever Management’ and five main categories. Nurses described challenges related to the absence of locally adapted clinical guidelines, lack of continuous education, hierarchical dependence on physicians, fever phobia and misconceptions, which led to non‐evidence‐based decision‐making and unnecessary interventions.

**Conclusion:**

The findings underscore the need for developing culturally appropriate local clinical guidelines, implementing evidence‐based continuous educational programmes, enhancing nurses' professional autonomy and providing effective family education. These interventions can improve care quality, reduce nurses' occupational anxiety and ultimately enhance child health outcomes.

**Implications for Profession and/or Patient Care:**

Understanding nurses' experiences and challenges may inform the development of educational interventions, localised clinical guidelines and organisational strategies to improve the quality and consistency of fever management in children.

**Impact:**

- What problem did the study address?
  ◦ The study addressed the gap between evidence‐based fever management guidelines and actual nursing practice in paediatric settings.
- What were the main findings?
  ◦ Nurses' clinical decision‐making was influenced by fear of complications, parental expectations, organisational factors and contextual factors.
- Where and on whom will the research have an impact on?
  ◦ The findings may impact paediatric nurses, healthcare administrators and policymakers by informing strategies to support evidence‐based fever management in hospital settings.

**Reporting Method:**

This study was reported in accordance with the Consolidated Criteria for Reporting Qualitative Research (COREQ) guidelines.

**Patient or Public Contribution:**

No patient or public contribution: Patients or members of the public were not involved in the design, conduct, reporting or dissemination plans of this research, as the study focused on nurses' professional experiences.

## 1 Introduction

Fever is one of the most common reasons for healthcare consultations and hospital admissions during childhood, with approximately 70% of preschool‐aged children experiencing at least one febrile episode each year (Milani et al. 2024). Although fever is generally a beneficial physiological response that enhances immune function and helps the body combat infection, it remains a major source of concern for both families and healthcare professionals. Persistent misconceptions about fever—commonly referred to as fever phobia—continue to influence healthcare‐seeking behaviours and clinical decision‐making despite decades of research and educational initiatives. As a result, many children undergo unnecessary medical evaluations, receive unnecessary or inappropriate antipyretic treatment and are exposed to avoidable healthcare interventions, imposing considerable psychological and economic burdens on families and healthcare systems (Merlo et al. 2023).

Beyond increasing healthcare utilisation, inappropriate fever management has important implications for medication safety and the quality of paediatric care. Current evidence consistently recommends that antipyretic medications should be administered to relieve a child's discomfort rather than to reduce body temperature alone. Nevertheless, antipyretics are still commonly prescribed or administered solely because a child has a fever, even in the absence of clinically significant distress. Such practices may contribute to medication errors, inaccurate dosing, unnecessary exposure to pharmacological interventions, adverse drug reactions and delayed recognition of the underlying causes of fever. Promoting evidence‐based fever management is therefore essential not only for improving clinical outcomes but also for ensuring safe, high‐quality paediatric care (Green, Krafft, et al. 2021; de Bont et al. 2020).

Paediatric nurses are central to fever management because they often spend more time with children and their families than any other healthcare professional during hospitalisation. Their role extends well beyond measuring body temperature or administering medications. Nurses are responsible for ongoing clinical assessment, monitoring changes in children's conditions, implementing both pharmacological and non‐pharmacological interventions, educating families, supporting parental decision‐making and alleviating anxiety related to childhood fever. Consequently, nurses' knowledge, beliefs, clinical judgement and communication skills substantially influence both the quality and consistency of fever management (Gaffney et al. 2021; Thompson et al. 2020).

## 2 Background

Although international clinical guidelines provide clear recommendations for evidence‐based fever management, considerable discrepancies remain between these recommendations and routine clinical practice. Studies from different healthcare settings have consistently shown that antipyretic medications continue to be administered more frequently than recommended, while non‐pharmacological strategies and family education are often underutilised (de Bont et al. 2020; Green, Webb, et al. 2021). These findings suggest that clinical decisions regarding childhood fever are influenced by factors that extend beyond scientific evidence alone.

Emerging evidence indicates that fever management is a complex clinical process shaped by psychological, social, organisational and cultural influences. Qualitative studies conducted in Switzerland and England have demonstrated that parental anxiety, expectations for rapid fever reduction and fear of complications frequently encourage unnecessary interventions and overtreatment (Merlo et al. 2023; Franklin et al. 2024). Similarly, research from Sweden has shown that physicians' decision‐making in the management of febrile infants is influenced not only by clinical findings but also by subjective judgement, professional experience and contextual pressures within healthcare settings (Orfanos et al. 2024). Together, these studies illustrate that evidence‐based recommendations are often interpreted and applied within broader cultural and organisational contexts.

Within the Iranian healthcare context, several studies suggest that misconceptions and anxiety surrounding childhood fever remain widespread among families. Research conducted among Iranian parents has reported persistent concerns about fever‐related complications, particularly febrile seizures, as well as deficiencies in knowledge, attitudes and practices related to fever management (Tavan et al. 2022). More recently, Soumei et al. (2026) found that a substantial proportion of Iranian parents perceived fever as harmful, reported high levels of anxiety when managing a febrile child and frequently associated fever with serious consequences such as febrile seizures and brain damage. Evidence from Iranian studies further indicates that febrile seizures remain one of the most common neurological conditions in childhood and an important source of parental concern, contributing to healthcare‐seeking behaviours and pressure for rapid fever reduction (Mohhamadi et al. 2023).

Collectively, these findings suggest that cultural beliefs, parental expectations and concerns about fever‐related complications continue to influence childhood fever management in Iran. Although previous Iranian research has primarily focused on parents' knowledge, attitudes, practices, educational interventions and the epidemiology of febrile seizures, comparatively little is known about how nurses experience childhood fever management in everyday clinical practice. Understanding these experiences is important because paediatric nurses play a central role in assessing febrile children, implementing therapeutic interventions, educating families and translating evidence into clinical practice. Exploring their perspectives may therefore provide valuable insights into the barriers and facilitators that influence evidence‐based fever management within the Iranian healthcare system.

To address this gap, we conducted a qualitative descriptive study using conventional content analysis to explore nurses' perceptions of childhood fever management and to gain a deeper understanding of the psychological, social, cultural and organisational factors influencing their clinical decision‐making, as well as the barriers and facilitators to evidence‐based practice.

## 3 The Study

### 3.1 Aim and Objectives

The aim of this study was to explore nurses' perceptions influencing their management of childhood fever, including the barriers and facilitators to evidence‐based practice.

Specifically, the study sought to:

- examine how nurses understand and interpret childhood fever;
- identify the psychological, social, cultural and organisational factors influencing their clinical decision‐making; and
- explore the barriers and facilitators to evidence‐based fever management in paediatric nursing practice.

## 4 Methods

### 4.1 Design

This study employed a qualitative descriptive design to explore nurses' perceptions and experiences of managing childhood fever in paediatric hospital settings. A qualitative descriptive approach was considered appropriate because little is known about how nurses make clinical decisions regarding childhood fever within the Iranian healthcare context, where cultural beliefs, organisational factors and interactions with families may influence everyday practice (Bradshaw et al. 2017). This design enabled a comprehensive description of participants' experiences using language that remained close to their own accounts while preserving the contextual complexity of clinical practice (Kim et al. 2017).

Data were analysed using an inductive qualitative content analysis approach as described by Graneheim and Lundman (2004).

The study was conducted and reported in accordance with the Consolidated Criteria for Reporting Qualitative Research (COREQ) checklist to enhance transparency, methodological rigour and completeness of reporting (Tong et al. 2007).

### 4.2 Study Setting

This study was conducted at Afzalipour Hospital, a tertiary referral teaching hospital affiliated with Kerman University of Medical Sciences. As a regional referral centre, the hospital provides a wide range of paediatric subspecialty services and receives referrals from across the province and neighbouring regions. The study was undertaken in the paediatric emergency department, three general paediatric wards, a paediatric haematology–oncology ward, neonatal intensive care units (NICUs) and paediatric intensive care units (PICUs). Recruiting participants from these diverse clinical settings enabled the inclusion of nurses with a broad range of experiences in managing childhood fever across different paediatric care contexts.

### 4.3 Participants and Sampling

Purposive sampling was used to recruit nurses who could provide rich descriptions of their experiences in managing childhood fever. Eligible participants were registered nurses working in the selected paediatric units who had at least one year of clinical experience, direct experience in caring for children with fever and were willing to share their professional experiences. Participants were free to withdraw from the study at any stage without providing a reason; however, no participants chose to withdraw.

After obtaining ethical approval from the Biomedical Research Ethics Committee of Kerman University of Medical Sciences and permission from the hospital administration, the first author met with the head nurses of the participating paediatric units to introduce the study. Eligible nurses were initially identified with the assistance of the head nurses based on the study inclusion criteria. Additional participants were then recruited purposively from different paediatric units to capture a broad range of clinical experiences related to childhood fever management. Potential participants were approached individually by the first author during visits to the clinical wards. The study aim, interview procedures, the voluntary nature of participation, confidentiality measures and participants' right to withdraw at any time without consequence were explained before written informed consent was obtained. Recruitment continued until data saturation was achieved. A total of 19 nurses were invited to participate, and all agreed to participate in the study.

### 4.4 Data Collection

Data were collected through individual semi‐structured interviews conducted between March 2024 and May 2025. The extended data collection period reflected the clinical nature of the study setting, as participants worked rotating shifts across different paediatric units. To minimise disruption to patient care, interviews were scheduled according to participants' availability, primarily during break times or after the completion of their work shifts. Data were collected by the first author, a PhD candidate in Paediatric Nursing and a member of the research team. The interviewer had no prior professional or personal relationship with the participants before the study commenced. Before each interview, the first author introduced herself, explained the purpose of the study and emphasised that participation was voluntary and that all information would remain confidential.

An interview guide was developed based on the study objectives and relevant literature. The guide was reviewed by faculty members with expertise in paediatric nursing and qualitative research to ensure the clarity, relevance and comprehensiveness of the interview questions. Examples of the interview questions are presented in Table 1.

**TABLE 1:** Examples of interview questions.

Can you describe your experience of caring for children with fever?
What challenges do you face in managing childhood fever in hospital wards?
In your opinion, which factors have the greatest influence on clinical decision‐making when managing childhood fever?
From your perspective, what role do families play in the management of childhood fever?
To what extent do existing protocols and guidelines assist you in managing childhood fever?

Interviews were conducted in a quiet and private location chosen by the participants, either in the staff break room or the education room within the paediatric ward. Most interviews took place during participants' break periods or after the completion of their clinical shifts.

Each interview lasted approximately 45–60 min and was audio‐recorded with participants' permission. Field notes were documented during and immediately after each interview to capture contextual observations and non‐verbal communication that informed the subsequent analysis.

Interviews began with broad, open‐ended questions, such as, ‘Can you describe your experience of caring for a child with fever?’ and ‘What challenges do you encounter when managing childhood fever in your clinical practice?’ Follow‐up and probing questions were used to clarify participants' responses and encourage further discussion when appropriate.

Interviews were transcribed verbatim shortly after completion and checked against the audio recordings for accuracy. Data collection continued until data saturation was achieved. Saturation was reached after the seventeenth interview, when no new codes or categories emerged from the data. Two additional interviews were conducted to confirm that no new insights were identified.

### 4.5 Data Analysis

The interview transcripts were analysed using the qualitative content analysis approach described by Graneheim and Lundman (2004). Each interview was transcribed before analysis, allowing coding to begin while data collection was still in progress. Data analysis was undertaken alongside data collection, allowing emerging findings to guide subsequent interviews and support the assessment of data saturation.

The analysis followed an inductive approach. Each transcript was read several times to achieve immersion in the data and gain an overall understanding of participants' experiences. Meaning units relevant to the study aim were identified, then condensed while preserving their essential meaning and assigned initial codes. Codes with similar meanings were compared and grouped into subcategories, which were subsequently organised into broader categories representing shared patterns across participants' accounts.

The first author conducted the coding using MAXQDA version 10. Throughout the analytic process, coding decisions and the category structure were regularly reviewed and discussed with other members of the research team to enhance the consistency and credibility of the analysis. Categories and interpretations were derived directly from the data rather than being guided by pre‐existing theoretical frameworks, ensuring that the findings closely reflected participants' perspectives and experiences. MAXQDA version 10 was used to organise, retrieve and manage the coded data throughout the analysis.

### 4.6 Ethical Considerations

This study was conducted in accordance with the ethical principles of the Declaration of Helsinki (World Medical Association, 2024). Ethical approval was obtained from the Biomedical Research Ethics Committee of Kerman University of Medical Sciences (Approval No. IR.KMU.REC.1403.354) before the study commenced.

Written informed consent was obtained from all participants before data collection. Participants received information about the study objectives, interview procedures, the voluntary nature of participation, potential benefits and risks, confidentiality measures and their right to withdraw from the study at any time without providing a reason or experiencing any consequences.

To protect participants' privacy, all interview transcripts were anonymised by assigning identification codes and removing any potentially identifiable information before analysis. Audio recordings and research data were stored securely and were accessible only to the research team. No financial or non‐financial incentives were provided for participation.

### 4.7 Rigour

The trustworthiness of the findings was established using the criteria proposed by Lincoln and Guba (1986), including credibility, transferability, dependability and confirmability.

Credibility was enhanced through prolonged engagement with the data, member checking and peer debriefing. Interview transcripts were returned to participants to verify that their experiences had been accurately represented and to provide an opportunity for clarification where needed. In addition, coding decisions and emerging categories were regularly discussed with members of the research team experienced in qualitative research to refine interpretations and strengthen the credibility of the analysis.

Transferability was supported by providing detailed descriptions of the study setting, participant characteristics, sampling strategy and data collection procedures, enabling readers to assess the applicability of the findings to similar contexts.

Dependability was strengthened by maintaining a clear audit trail documenting the research process, including methodological decisions, interview procedures, coding and category development.

Confirmability was enhanced through reflexive notes recorded throughout the study to encourage ongoing reflection on the researcher's assumptions and their potential influence on data interpretation. The analytic process and coding decisions were also reviewed with members of the research team to promote transparency and minimise individual researcher bias.

## 5 Findings

### 5.1 Participant Characteristics

Participant characteristics are presented in Table 2. Nineteen paediatric nurses participated in the study, representing a range of clinical roles across paediatric wards, the paediatric intensive care unit (PICU), the neonatal intensive care unit (NICU), the paediatric emergency department and the paediatric haematology–oncology ward. Participants varied in age, educational background and years of clinical experience, providing diverse perspectives on childhood fever management.

**TABLE 2:** Demographic and professional characteristics of the participants (*n* = 19).

Characteristic	*n* (%) or mean ± SD (range)
Gender	
Female	13 (68.4)
Male	6 (31.6)
Highest educational qualification	
Bachelor's degree	11 (57.9)
Master's degree	8 (42.1)
Age (years)	34.1 ± 6.2 (25–45)
Years of paediatric nursing experience	10.3 ± 5.4 (2–20)
Clinical setting	Paediatric ward, PICU, NICU, Paediatric emergency department, Paediatric haematology–oncology ward

### 5.2 The Main Theme and Categories

Analysis of the interview data identified one overarching theme, Navigating Uncertainty and Psychological Strain in Paediatric Fever Management, together with five main categories and 12 subcategories (Table 3). The overarching theme captures nurses' experiences of managing childhood fever while balancing clinical uncertainty, emotional demands, organisational challenges and interactions with families. The five categories and their corresponding subcategories are presented in Table 3. The findings are presented below and illustrated with representative quotations from the participants.

**TABLE 3:** The main themes, categories, subcategories and codes.

Main theme	categories	Subcategories	codes
Navigating uncertainty and psychological strain in paediatric fever management	Ambiguity and inconsistency in guideline‐based practice	Lack of clear and accessible clinical guidance	To have unclear unit guidelines, leading to varying choices in fever treatment To lack specific instructions, resulting in diverse fever‐care behaviours To rely on personal experience in fever management when guidelines are unavailable To be unfamiliar with existing guidelines, limiting consistency in nursing practice To have inadequate knowledge of clinical guidelines, affecting guideline‐informed care
		Guidelines: The overlooked imperative	To increase nurses' confidence in fever control through guideline use To promote consistent fever management practices among nursing staff To foster integration of doctor–nurse performance via shared clinical guidance To facilitate uniform fever‐treatment decisions among physicians To prevent arbitrary actions through standardised care To enhance parental trust in care through evidence‐based guidelines To support orientation and training of new staff via accessible clinical guidance
	Educational gaps and need for continuous professional development	Limited training opportunities	To have limited continuing education programmes for nurses on fever management To experience insufficient opportunities to attend current fever‐focused training workshops To receive minimal new education on emerging fever treatment approaches
		Need for evidence‐based knowledge updates	To update fever‐management knowledge based on current evidence To prioritise updating clinical education with current guideline developments
		Challenges in family education and communication	To be unable to educate families effectively about fever physiology and management To misinterpret warning signs due to insufficient parent education on fever severity
	Experiential practice and dependence on physician authority	Balancing experiential and scientific knowledge	To rely on personal experience in fever management in the absence or contrast of scientific guidance To integrate experiential knowledge and research‐based knowledge in fever care as complementary approaches To increase accuracy in fever management through accumulated nursing experience
		Dependence on physician authority	To rely on physicians' instructions when selecting antipyretic medications To rely on physicians' instructions when determining fever‐management approaches To depend more on physicians' guidance and make fewer autonomous decisions in fever care
	Fear‐driven and anxiety‐based practices	Fear of fever complications	Fearing febrile seizures when a child has fever To fear inadequate fever control might lead to child's condition worsening To perceive sudden high fever in a child as a danger requiring urgent response To believe fever could cause brain damage in children To experience doubt and anxiety when managing paediatric fever as a novice nurse
		Psychological distress during ineffective interventions	To experience elevated stress when fever‐reducing interventions appear ineffective To prompt rapid fever control without investigating cause due to seizure concern To experience greater fear following previous negative fever‐related events To anticipate recurrence of past adverse events when dealing with current fever
	Beliefs and misconceptions about childhood fever management	Misconceptions about fever management	To consider non‐pharmacological methods ineffective To doubt the effectiveness of common fever‐control methods To experience uncertainty about the response to antipyretic drugs in some children To repeat treatments in an attempt to reduce anxiety for oneself or the family
		Fear of judgement and external pressure	To anticipate judgement from physicians or family if fever management is perceived inadequate To experience heightened anxiety when fever remains elevated and treatment effectiveness is uncertain To interpret fever presence as inherently dangerous

#### 5.2.1 Category 1: Ambiguity and Inconsistency in Guideline‐Based Practice

Participants consistently described the absence of locally adapted clinical guidelines as one of the main challenges in managing childhood fever. They explained that the lack of standardised guidance resulted in variations in clinical practice, uncertainty in decision‐making and inconsistent approaches among healthcare professionals. This category comprises two subcategories.

##### 5.2.1.1 Subcategory 1–1: Lack of Clear and Accessible Clinical Guidance

Many participants explained that, in the absence of clear and accessible evidence‐based guidelines, they often relied on personal clinical experience or physicians' preferences when managing childhood fever. As a result, approaches to care varied across clinicians and clinical situations.

We don't have any specific guidelines for managing fever; we usually just go with our own experience. (P3)

I wish there was a single, uniform guideline so we wouldn't have to keep relying on physicians' opinions—every doctor has a different view. (P5)

Each physician has their own method: one jumps straight to medication, while another starts with non‐pharmacological approaches. (P1)

##### 5.2.1.2 Subcategory 1–2: Guidelines: The Overlooked Imperative

Participants emphasised the importance of developing evidence‐based clinical guidelines that are relevant to the local healthcare context. They believed that standardised guidance would strengthen clinical confidence, promote greater consistency in practice, facilitate the orientation of newly employed nurses and improve communication among members of the healthcare team.

Families trust us when they see confidence in the staff, and we feel confident only when we have a standard guideline. (P9)

Guidelines would make training new staff much easier. (P12)

Several participants explained that receiving different advice from healthcare professionals often created confusion for both nurses and families.

One day, the mother of a sick child told me, ‘Why does everyone say something different?’ I felt so confused… That moment made me realize how much we're missing a standard guideline. (P15)

We don't have guidelines in Iran, but it would be great if we did—no one could find fault, your conscience would be clear, and new nurses could work without stress. (P11)

If we had clear protocols, we wouldn't need to ask the physician every time or wait for orders; we could act confidently on our own. (P8)

#### 5.2.2 Category 2: Educational Gaps and Need for Continuous Professional Development

Participants consistently described limited educational opportunities as an important challenge in the management of childhood fever. They believed that insufficient access to structured education and evidence‐based updates affected their confidence in clinical decision‐making and contributed to variations in practice. This category comprises three subcategories.

##### 5.2.2.1 Subcategory 2–1: Limited Training Opportunities

Participants described limited opportunities for continuing professional education on childhood fever management. Many explained that their clinical knowledge relied largely on what they had learned during undergraduate education or through personal reading rather than structured in‐service training.

Fever management training isn't part of the hospital's educational programs, or it's rarely offered. (P4)

Everything I know about fever control comes from what I learned as a student. (P10)

Several participants explained that, in the absence of formal educational programmes, they relied on self‐directed learning to update their knowledge.

We don't have any official educational resources in the ward—we just share whatever we read ourselves with the team. (P6)

It would be wonderful if practical workshops were held for real scenarios, like dealing with resistant fever or managing parental anxiety. (P2)

In‐service training is extremely rare, and when it does happen, it's purely theoretical. Adding practical exercises would make a big difference. (P11)

##### 5.2.2.2 Subcategory 2–2: Need for Evidence‐Based Knowledge Updates

Participants emphasised the importance of regularly updating their knowledge in line with current evidence and clinical guidelines. They believed that ongoing education would strengthen clinical decision‐making, increase confidence and support more consistent evidence‐based care.

If training were based on the latest research, our decisions would be more scientific and accurate. (P5)

Knowing the most recent recommendations would really boost our confidence and help us make fewer mistakes. (P18)

Several participants explained that although they tried to update their knowledge independently, they believed that self‐directed learning could not replace organised educational programmes.

I sometimes read articles on my own or verify what physicians say against reliable sources, but formal, structured training is much more effective and ensures everyone gets the same information. (P12)

Ongoing education is essential to stay aligned with new standards; otherwise, we just keep following old habits. (P17)

Up‐to‐date training would help us avoid unnecessary interventions and provide truly evidence‐based care. (P10)

##### 5.2.2.3 Subcategory 2–3: Challenges in Family Education and Communication

Participants described family education as an important but challenging aspect of childhood fever management. They explained that many parents had limited knowledge about fever and often viewed it as a dangerous condition requiring immediate treatment. As a result, nurses spent considerable time addressing parents' concerns, explaining clinical decisions and providing education during routine care.

…parents believe that every fever needs to be treated quickly because they're afraid of its complications. (P11)

Mothers don't know exactly what to do when their child has a fever or how to manage it—this stems from not receiving proper education. (P18)

They need to be educated in a way that allows them to actually apply it during caregiving, but in reality, that's not how it is. (P16)

We nurse and doctors should provide education that's tailored to the mothers' level of understanding, but in practice, that's not the case. (P15)

#### 5.2.3 Category 3: Experiential Practice and Dependence on Physician Authority

Participants described relying heavily on clinical experience and physicians' decisions when managing childhood fever, particularly in situations where clear clinical guidelines were unavailable. They explained that everyday clinical practice often involved balancing personal experience with scientific knowledge while working within a hierarchical healthcare system. This category comprises two subcategories.

##### 5.2.3.1 Subcategory 3–1: Balancing Experiential and Scientific Knowledge

Participants viewed clinical experience as an important resource in the management of childhood fever, particularly when clear clinical guidelines were unavailable. At the same time, they emphasised that clinical experience should be complemented by current scientific evidence to support safe and consistent decision‐making.

Experience is valuable—as long as it's integrated with evidence. (P3)

Without standard protocols … experience unconsciously leads us to make individual decisions. (P1)

Experience can be extremely helpful, but without scientific evidence, we might make mistakes. (P15)

Several participants also reflected on the challenges of balancing their own clinical experience with current evidence in everyday practice.

In my opinion, personal experiences are an inseparable part of individual professional skills. (P19)

Personal experience and existing knowledge don't always match. (P10)

##### 5.2.3.2 Subcategory 3–2: Dependence on Physician's Authority

Participants described that decisions related to childhood fever management were often influenced by physicians' preferences and treatment decisions. Many explained that, particularly in complex clinical situations, they waited for physicians' instructions before initiating specific interventions.

Usually in critical situations … we wait for the physician's instructions. (P2)

Sometimes I feel we have no independence at all—we're merely carrying out physicians' orders. (P5)

Physicians' opinions vary … for providing care, we have to see which physician the patient is assigned to. (P7)

#### 5.2.4 Category 4: Fear‐Driven and Anxiety‐Based Practices

Participants described fear and anxiety as important influences on their clinical decision‐making when caring for children with fever. Many explained that concerns about potential complications and the emotional demands of caring for febrile children affected how they assessed situations and responded in clinical practice. This category comprises two subcategories.

##### 5.2.4.1 Subcategory 4–1: Fear of Fever Complications

Participants frequently described concerns about serious complications associated with fever, particularly febrile seizures. They explained that previous clinical experiences, personal beliefs and the responsibility of caring for children contributed to these concerns and influenced their responses to febrile episodes.

…Every time a child has fever, I worry they might have a seizure. (P6)

I usually start treatment quickly out of fear of fever complications. (P11)

I've cared for a child admitted due to high fever… and I'm always afraid of that complication. (P13)

When a child's fever rises and doesn't come down, I get stressed—I keep thinking, ‘What if they seize now?’ (P12)

##### 5.2.4.2 Subcategory 4–2: Psychological Distress During Ineffective Interventions

Participants described considerable psychological distress when fever persisted despite treatment. They explained that ongoing fever often increased anxiety for both families and healthcare professionals and created additional pressure during clinical decision‐making.

When the fever doesn't come down, …I feel like everything we've done has been pointless. (P8)

Sometimes we're forced to start drug therapy just to calm the family down. (P1)

Because fever makes the child restless, and then the mother becomes restless too… we're compelled to initiate pharmacological treatment quickly, even if it's not necessary. (P7)

Controlling fever has a lot to do with psychological aspects—maintaining calmness for yourself, the mother, and the child is very important. (P12)

#### 5.2.5 Category 5: Beliefs and Misconceptions About Childhood Fever Management

This category reflects nurses' beliefs and assumptions about childhood fever and its management, as well as the influence of external expectations on clinical decision‐making. Participants described uncertainty regarding the effectiveness of different fever‐management strategies, limited confidence in non‐pharmacological approaches and concerns about how their actions might be perceived by families and other healthcare professionals. Together, these factors often contributed to early pharmacological intervention and departures from evidence‐based practice. The category comprised two subcategories.

##### 5.2.5.1 Subcategory 5–1: Misconceptions About Fever Management

Participants described uncertainty regarding the effectiveness of different fever‐management strategies and often expressed limited confidence in non‐pharmacological approaches. Several nurses believed that fever could not be managed effectively without pharmacological treatment and questioned whether commonly recommended non‐pharmacological interventions were sufficient to control fever. These perceptions frequently led to the early administration of antipyretic medications and repeated interventions when fever persisted.

…We try non‐pharmacological methods, but since we're not confident that they're effective on their own, we quickly start pharmacological treatment as well. (P10)

…And most of the time, I quickly move to the pharmacological phase. (P12)

…I don't have much confidence in tepid sponging or other non‐pharmacological interventions for managing fever. (P18)

Participants also described uncertainty about how individual children might respond to fever treatments. When fever did not decrease as expected, some reported feeling compelled to repeat interventions or escalate treatment in an effort to reduce their own concerns and reassure families.

##### 5.2.5.2 Subcategory 5–2: Fear of Judgement and External Pressure

Participants described feeling pressure from parents, physicians, colleagues and the broader healthcare environment when caring for children with fever. They explained that concerns about being judged, together with parents' expectations for rapid fever reduction, often influenced their clinical decisions.

If the fever doesn't come down, parents might think that I don't know what I'm doing or that I've been negligent. (P11)

Fever is like an alarm bell—everyone expects it to be treated quickly, even if the child isn't uncomfortable. (P10)

…With a mother who isn't very alert or has lower literacy, no matter how much I explain … she won't accept it until we give medication. (P13)

It's about priorities and the family's emotional state … sometimes we start medication early just to calm the mother down. (P7)

## 6 Discussion

This study provides insight into the complex factors influencing paediatric nurses' management of childhood fever within the Iranian healthcare system. The findings suggest that nurses' clinical decision‐making is shaped not only by their knowledge of evidence‐based recommendations but also by organisational structures, educational opportunities, professional relationships, psychological responses and culturally embedded beliefs about fever. Together, these factors contributed to an overarching experience of uncertainty in everyday clinical practice, often making it difficult for nurses to consistently implement evidence‐based fever management.

The findings suggest that the absence of locally adapted guidelines creates uncertainty not only about clinical decision‐making but also about professional accountability. In this context, nurses appeared to rely on personal experience and physicians' preferences to compensate for the lack of standardised guidance, which may contribute to variability in care for childhood fever management. Similar inconsistencies have been reported internationally. Gaffney et al. (2021) found considerable variation in nurses' fever management practices despite the availability of international recommendations, while Green, Krafft, et al. (2021) demonstrated substantial discrepancies among national and international fever guidelines. However, the present findings suggest that the challenge extends beyond guideline availability. Participants emphasised that clinical guidelines must also be adapted to local healthcare systems and clinical realities if they are to support nurses' decision‐making effectively. This finding is particularly relevant in healthcare environments where organisational structures, professional hierarchies and cultural expectations influence everyday clinical practice. Developing context‐specific, evidence‐based guidelines may therefore improve consistency of care while strengthening nurses' confidence and interdisciplinary communication.

Another important finding concerned the limited opportunities for continuing professional education. Although participants recognised the importance of evidence‐based practice, many reported that their knowledge of childhood fever management relied largely on undergraduate education or self‐directed learning. Similar educational gaps have been identified in previous studies. A systematic review by Vicens‐Blanes et al. (2021) concluded that insufficient knowledge remains a common barrier to evidence‐based fever management among healthcare professionals. Likewise, Milani et al. (2023) reported persistent misconceptions regarding fever physiology among nursing students despite formal education. The present study extends these findings by showing that nurses themselves perceive continuing professional education as essential for maintaining clinical competence in rapidly evolving practice environments. Participants consistently viewed structured educational programmes as an opportunity not only to update scientific knowledge but also to strengthen clinical confidence and reduce variations in practice. These findings support the integration of regular evidence‐based educational initiatives into professional development programmes for paediatric nurses.

Participants also described how interactions with families represented an important component of fever management. Nurses frequently encountered parents who viewed fever as inherently dangerous and expected immediate intervention regardless of the child's overall clinical condition. Responding to these expectations often required considerable time, communication and emotional effort. Similar observations have been reported in previous studies demonstrating that parental anxiety and misconceptions about fever continue to influence healthcare utilisation and clinical decision‐making (Merlo et al. 2023; Franklin et al. 2024). The findings suggest that improving parental education should be considered an integral component of evidence‐based fever management rather than an activity separate from clinical care. Equipping nurses with effective communication skills alongside updated clinical knowledge may therefore facilitate shared decision‐making and reduce unnecessary interventions.

Another notable finding was nurses' reliance on physicians' decisions when managing childhood fever. Participants described situations in which uncertainty, together with the absence of standardised protocols, limited their professional autonomy and resulted in delays while awaiting medical orders. Although collaborative decision‐making is fundamental to paediatric care, participants perceived that hierarchical relationships sometimes reduced opportunities to exercise independent clinical judgement. Similar concerns have previously been described by Purssell (2014), who highlighted the influence of organisational culture on nursing decision‐making in fever management. The present findings suggest that strengthening evidence‐based nursing protocols may support greater professional autonomy while promoting more consistent interdisciplinary care.

Psychological factors also emerged as an important influence on clinical decision‐making. Many nurses described persistent concerns about febrile seizures and other potential complications, particularly when fever did not respond rapidly to treatment. These concerns frequently led to conservative clinical decisions and early use of antipyretic medications, even when children showed relatively mild symptoms. Previous studies have similarly demonstrated that fever phobia extends beyond parents to healthcare professionals and may contribute to overtreatment (Merlo et al. 2023; Gaffney et al. 2021; Bertille et al. 2016). The present findings further illustrate how emotional responses, previous clinical experiences and perceived professional responsibility interact to influence decision‐making in everyday practice. Addressing these psychological influences may therefore be as important as improving clinical knowledge when promoting evidence‐based fever management.

Finally, participants reported several misconceptions related to fever management, including limited confidence in non‐pharmacological approaches, uncertainty regarding treatment effectiveness and concerns about being judged by families or colleagues when fever persisted. These beliefs appeared to reinforce unnecessary pharmacological interventions and contributed to variations in practice. Previous research has similarly shown that misconceptions about fever remain common among healthcare professionals despite the increasing availability of clinical evidence (Gaffney et al. 2021; Thompson et al. 2020). Our findings suggest that correcting misconceptions requires more than simply disseminating guidelines. Educational interventions should also address underlying beliefs, clinical confidence and the organisational culture in which fever management takes place.

Overall, the findings demonstrate that paediatric fever management is influenced by an interplay of clinical, educational, organisational, psychological and cultural factors. While many of these challenges have been reported internationally, the present study highlights how they interact within the Iranian healthcare context to shape nurses' everyday clinical practice. Addressing these interconnected factors through locally adapted clinical guidelines, ongoing professional education, organisational support and family‐centred communication may facilitate more consistent implementation of evidence‐based fever management and ultimately improve the quality of paediatric care.

### 6.1 Strengths and Limitations of the Work

A major strength of this study is the in‐depth exploration of paediatric nurses' lived experiences using a rigorous qualitative content analysis approach. The inclusion of nurses from diverse paediatric units enhanced the richness and credibility of the data.

However, the study was conducted in a single hospital setting, which may limit the transferability of the findings to other contexts. Future studies across multiple institutions may provide broader insight into paediatric fever management practices.

### 6.2 Recommendations for Further Research

Future research should examine paediatric fever management across different healthcare settings and cultural contexts to better understand systemic influences. Intervention studies may also be valuable to assess the effectiveness of educational programmes, evidence‐based guideline implementation and communication strategies aimed at reducing fever phobia among both healthcare professionals and families. Mixed‐methods approaches could further validate and expand upon the qualitative insights identified in this study.

## 7 Conclusion

This study highlights how organisational, educational, professional, psychological and cultural factors interact to shape paediatric nurses' management of childhood fever. Although many of these challenges have been reported in other healthcare settings, the findings suggest that, within the Iranian context, the absence of locally adapted clinical guidelines, limited opportunities for continuing professional education, hierarchical decision‐making structures and persistent misconceptions about fever contribute to uncertainty in everyday clinical practice. This study contributes to the limited qualitative evidence on paediatric fever management in Iran by demonstrating how these interconnected factors collectively influence nurses' clinical decision‐making and implementation of evidence‐based care. Improving childhood fever management requires a multifaceted approach. Developing context‐specific evidence‐based clinical guidelines, providing ongoing professional education, strengthening nurses' confidence and autonomy in clinical decision‐making and supporting effective communication with families may promote more consistent implementation of evidence‐based fever management. In addition, improving family education may help reduce parental anxiety, facilitate collaboration between families and healthcare professionals and ultimately improve the quality of paediatric care.

Future research should evaluate the implementation and effectiveness of locally adapted clinical guidelines and educational interventions across different healthcare settings and examine their impact on nurses' clinical practice, family experiences and child health outcomes.

## Author Contributions

Conceptualisation: Zahra Sarkoohi, Monirsadat Nematollahi, Jamileh Farokhzadian, Maryam Askaryzadeh Mahani. Methodology: Zahra Sarkoohi, Monirsadat Nematollahi, Jamileh Farokhzadian. Formal Analysis: Zahra Sarkoohi, Monirsadat Nematollahi. Investigation: Zahra Sarkoohi. Data Curation: Zahra Sarkoohi. Writing – Original Draft: Zahra Sarkoohi. Writing – Review and Editing: Monirsadat Nematollahi, Jamileh Farokhzadian, Maryam Askaryzadeh Mahani. Supervision: Monirsadat Nematollahi.

## Funding

The authors have nothing to report.

## Ethics Statement

This study was conducted in accordance with the ethical principles of the Declaration of Helsinki (World Medical Association, 2024). The study was approved by the Ethics Committee of Kerman University of Medical Sciences (Approval No. IR.KMU.REC.1403.354). Before conducting the study, the researcher obtained informed consent from the nurses and ensured the confidentiality of the responses. Consent included permission for audio‐recording and the anonymised use of direct quotations in publications. Participation was entirely voluntary, and nurses were assured that refusal or withdrawal would have no impact on their employment status. All interviews were de‐identified, securely stored and accessible only to the research team. In addition, the study was conducted in accordance with internationally recognised standards for research integrity and publishing ethics, including the Wiley Publishing Ethics Guidelines.

## Consent

All participants provided consent for the anonymised use of interview data and direct quotations in publications.

## Conflicts of Interest

The authors declare no conflicts of interest.

## Data Availability

Due to the qualitative nature of this study and the need to protect participant confidentiality, the interview transcripts are not publicly available. De‐identified data may be made available from the corresponding author upon reasonable request.
